# CSF disturbances and other neurosurgical complications after interdisciplinary reconstructions of large combined scalp and skull deficiencies

**DOI:** 10.1007/s10143-020-01347-7

**Published:** 2020-07-10

**Authors:** Vicki M. Butenschoen, Jochen Weitz, Lucas M. Ritschl, Bernhard Meyer, Sandro M. Krieg

**Affiliations:** 1 School of Medicine, Department of Neurosurgery, Klinikum rechts der Isar, Technical University Munich, Ismaninger Str. 22, 81675 Munich, Germany; 2 School of Medicine, Department of Oral and Maxillofacial Surgery, Klinikum rechts der Isar, Technische Universität München, Ismaningerstr. 22, 81675 Munich, Germany

**Keywords:** Skull defects, Interdisciplinary treatment, Neurological complications

## Abstract

Combined scalp and skull deficiency due to malignant scalp tumors or sequelae of intracranial surgery present challenging entities for both neurosurgeons and reconstructive treatment. In complex cases, an interdisciplinary approach is needed between neurosurgeons and cranio-maxillofacial surgeons. We present a considerably large series for which we identify typical complications and pitfalls and provide evidence for the importance of an interdisciplinary algorithm for chronic wound healing complications and malignomas of the scalp and skull. We retrospectively reviewed all patients treated by the department of neurosurgery and cranio-maxillofacial surgery at our hospital for complex scalp deficiencies and malignant scalp tumors affecting the skull between 2006 and 2019, and extracted data on demographics, surgical technique, and perioperative complications. Thirty-seven patients were treated. Most cases were operated simultaneously (*n*: 32) and 6 cases in a staged procedure. Nineteen patients obtained a free flap for scalp reconstruction, 15 were treated with local axial flaps, and 3 patients underwent full thickness skin graft treatment. Complications occurred in 62% of cases, mostly related to cerebrospinal fluid (CSF) circulation disorders. New cerebrospinal fluid (CSF) disturbances occurred in 8 patients undergoing free flaps and shunt dysfunction occurred in 5 patients undergoing local axial flaps. Four patients died shortly after the surgical procedure (perioperative mortality 10.8%). Combined scalp and skull deficiency present a challenging task. An interdisciplinary treatment helps to prevent severe and specialty-specific complications, such as hydrocephalus. We therefore recommend a close neurological observation after reconstructive treatment with focus on symptoms of CSF disturbances.

## Introduction

Large skin defects due to chronic wound healing disorders and scalp deficiency, as well as scalp and skull malignancies such as spinalioma and metastases, present a challenge for health care providers [4, 26]. In some cases, patients need to undergo multiple surgeries, and an interdisciplinary approach is needed to provide a sufficient and esthetically satisfying scalp and skin reconstruction [21, 22].

In cases of large scalp defects, different methods can be applied to overcome scalp deficiency. First, local flaps can be used to cover small scalp defects [28], then, microvascular free flaps [15] such as the latissimus dorsi flap, vastus lateralis flap [11], or (para-) scapular flap, are used for reconstruction. In cases of the mentioned microvascular free flaps, donor sites are closed by primary wound closure. Free flaps for scalp reconstruction are considered a safe and successful procedure in complex and challenging cases of scalp and calvarial defects [12]. In terms of skull defects, such as in patients with chronic wound healing disorders and infected or osteolytic calvarial bone, a cranioplasty with alloplastic material is planned to restore cosmesis [1, 13, 25].

The literature focusing on scalp reconstruction is mainly determined by cases and techniques reported by craniofacial surgeons, despite the patient population being neurosurgical cases with initial diagnoses treated in neurosurgical departments.

In our study, we aim to analyze cases treated in an interdisciplinary way, including the neurosurgical and cranio-maxillofacial surgery department, to identify potential risk factors in patients with complicated scalp and skull defects.

## Material and methods

### Patients

All patients treated in an interdisciplinary way between 2006 and 2019 by the neurosurgical and cranio-maxillofacial surgery departments at our clinic were retrospectively screened for procedures performed due to large chronic wound healing disturbances or malignomas with the need for local or free flap reconstruction of the scalp and skull (inclusion criteria: data on operative therapy available, explicit diagnosis stated, follow-up data complete).

**Fig. 1:**
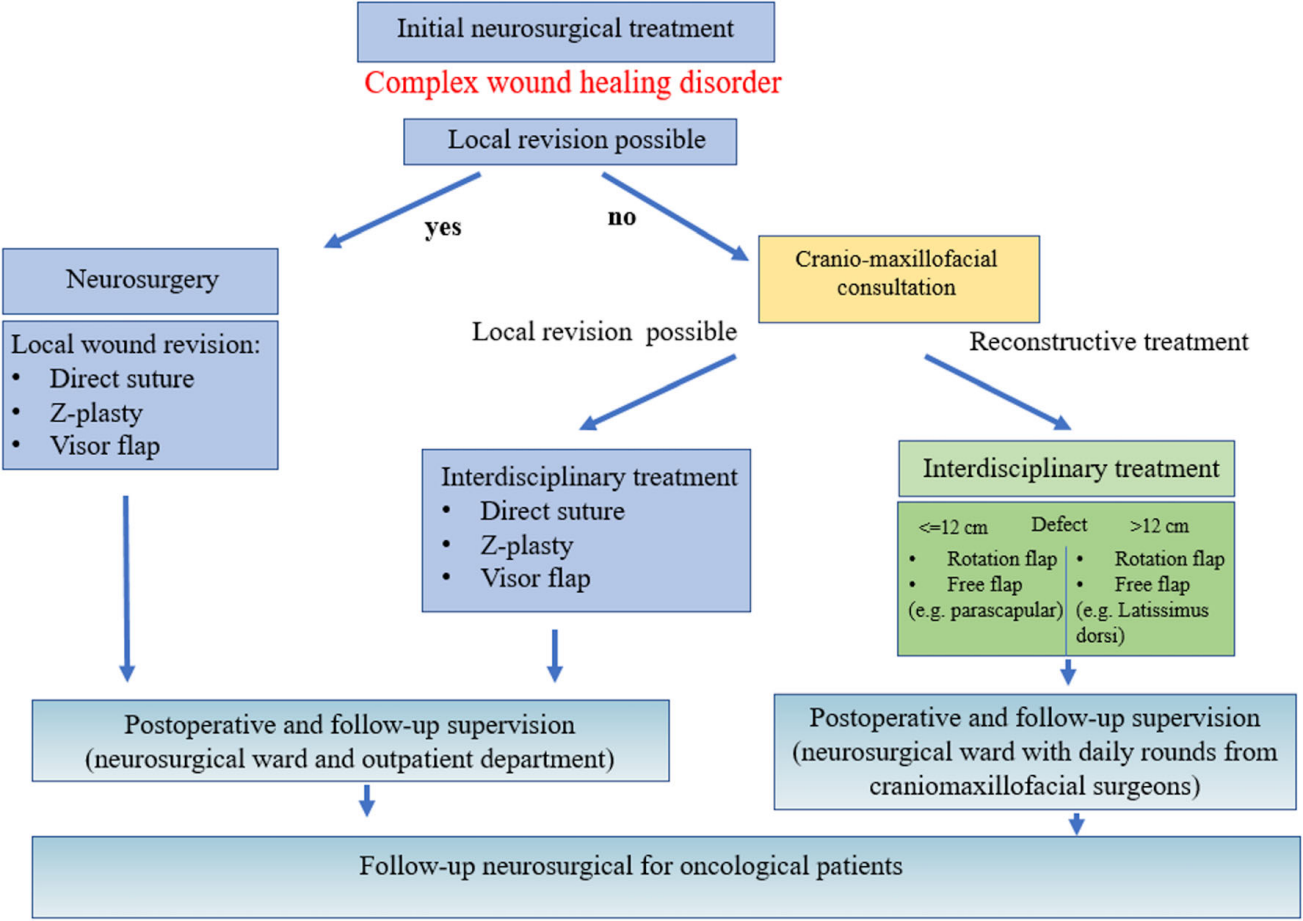
Proposed algorithm for the interdisciplinary treatment of neurosurgical patients undergoing reconstructive surgery

Information on a patient’s demographics, as well as detailed data on the surgical performance and technique, duration of surgery, perioperative complications and management, as well as length of hospital stay were retrieved from our archives and compared depending on the surgical procedures performed in terms of surgical and neurological outcome (Fig. 1).

### Ethics

The present study is in accordance with ethical standards outlined in the Declaration of Helsinki; the ethics approval was obtained on the 28th of May 2018 and is listed under document number 218/18 S.

### Statistics

Statistical analyses were performed using the Excel spreadsheet statistics software with data analysis package (Microsoft) and SPSS (IBM Corporation Version 26). The statistical hypothesis test was run by a two-sided *t* test following the Student’s *t* distribution for paired samples. A *p* value < 0.05 was considered significant. We performed bivariate and multivariate analysis to analyze possible influencing factors. Multivariate variance analysis was performed with ANOVA.

## Results

### Patients

In total, 37 patients matched our previously defined inclusion criteria. Fifty-one percent of the patients were female (19/37) and 49% were male (18/37). Median age at time of surgery was 56 years (range 26–86 years). Eighty-four percent of the patients were treated in an interdisciplinary way in a simultaneous operative procedure (neurosurgical intervention and reconstructive surgery, 31 cases), and in 16% of the cases (6/37), a staged procedure was preferred with a delay of 1 to 45 days (mean 17 days).

In total, 11 patients (29.7%) underwent a surgical treatment for hydrocephalus or cerebrospinal fluid (CSF) circulation disorder (ventriculo-peritoneal VP shunt) prior to the scalp and skull reconstruction.

### Medical history

The main initial diagnoses leading to the first operative neurosurgical treatment were recurrent high-grade astrocytoma and traumatic brain injury, followed by recurrent meningioma. The numbers are given in Table 1. In 3 cases, skin tumors of the scalp and the skull were surgically removed (1 basalioma, 2 spinalioma) (Table 1).

**Table 1:** Causes of initial craniotomy

Causes of initial craniotomy	
SAH	16%
GBM	27%
Hematoma	27%
Meningeoma	22%
Skin tumor	8%
Total	100%

The leading cause was primary or recurrent glioblastoma (GBM, 27%) and surgically treated hematoma (27%) followed by meningioma (22%), subarachnoid hemorrhage (16%) and skull infiltrating skin tumors (8%)

On average, patients with chronic wound healing disorders underwent a median of 4 surgeries (range 2 to 12) before the interdisciplinary scalp reconstruction was performed; patients with recurrent or primary tumor lesions underwent a median of 3 surgeries (range 0 to 5). The average duration between the initial cranial operative treatment and the need for interdisciplinary wound treatment was 224 days (range 0 to 41.6 years).

### General procedure

Thirty-seven patients were treated in total. In 31 patients, the surgical therapy was performed in an interdisciplinary way simultaneously with both neurosurgical and craniofacial expertise in one operative step. In 6 patients, the surgical procedure was staged, with a neurosurgical intervention performed prior to the flap transfer and wound closure (with a time interval of 31 days prior to 45 days after reconstructive therapy).

### Neurosurgical procedure

Neurosurgical performances included the treatment of former cranioplasties, either the implantation with computer-aided design (CAD) titanium implants (5/37) or autologous bone graft (2/37) or the removal of the affected cranioplasty (7/37), indicating the need for reconstructive treatment of the osseous compartment and/or dura. In 5 patients, a previously implanted shunt was replaced/transferred to another location (5/37). Ten patients underwent tumor resection and 2 patients were operated for a hematoma. Four patients had their autologous bone graft reduced and 2 patients underwent wound debridement with subdural inspection.

Mean duration of the interdisciplinary surgery was 232 min (range 25 to 672 min).

### Scalp reconstruction

Different approaches were applied to patients presenting with scalp deficiencies. Only complex composite defects were included; local wound revisions were excluded due to their missing complexity and interdisciplinary treatment. Local transposition flaps were applied in 15 out of 37 cases and free flaps in 19 complex scalp defects, including the transfer of a latissimus dorsi muscle flap, scapular and parascapular muscle flaps, radial forearm, and the anterolateral thigh (ALT) flap. In one patient, a tissue expander was implanted prior to the reconstruction (Table 2).

**Table 2:** Reconstructive technique used during surgery, most patients were treated with local axial flaps (15 cases, 41%) or anterolateral thigh flap (ALT) (6 cases, 16%)

Reconstructive technique	%	*n*	Mean duration (min)	Range (min)
Local axial flap	41	15	93	25–204
Latissimus dorsi flap	14	5	357	119–555
Parascapular flap	14	5	387	91–672
Radial forearm flap	8	3	436	359–533
ALT	16	6	297	159–500
Full-thickness skin graft	8	3	75	30–165
Total	100	37	232	25–672

Mean duration ranged from 75 min (full thickness skin graft) to 436 min (radial forearm flap)

In total, in 29 patients (74%) postoperative intravenous antibiotics were administered.

### Outcome and complications

Complications occurred in 62% of the cases (22/37). Minor complications included CSF leaks (*n*: 1) or urinary tract infections with the need of intravenous antibiotic treatment (13/37) (Table 3).

**Table 3:** Complications occurring after reconstructive grouped by reconstructive technique

	Local axial flap n (%)	Free flap *n* (%)	Full thickness graft *n* (%)	Total *n* (%)
Number of patients	15 (41)	19 (51)	3 (8)	37 (100)
Preoperative Shunt	8 (53)	3 (16)	0 (0)	11 (30)
Complications	9 (60)	11 (58)	2 (66)	22 (59)
Minor complications (UTI)	8 (53)	5 (26)	0 (0)	13 (35)
Minor complications (ventriculitis)	3 (20)	4 (21)	0 (0)	7 (19)
CSF disturbances (new)	0 (0)	8 (42)	1 (33)	9 (24)
Shunt dysfunction	5 (33)	0 (0)	0 (0)	5 (14)
Hemorrhage	1 (7)	2 (10)	0 (0)	3 (8)
Flap revision	2 (13)	0 (0)	1 (33)	3 (8)
Mortality	0 (0)	4 (21)	0 (0)	4 (11)

Remarkably, many patients suffered from postoperative CSF disturbances in patients undergoing free flap transfer (42%, *n*: 8). Shunt dysfunction occurred in a third of the patients treated with local axial flap technique. Overall mortality was 11%

Wound healing problems continued to occur in 5 patients (12.8%) with a need for further operative treatment (in 3 cases a new flap was transferred, 2 cases were treated with local wound adaptations).

In 40.5% of the cases (15/37), patients suffered from a postoperative CSF circulation disorder and 4 underwent surgery for VP shunting after scalp reconstruction after a median of 6 days (range 1 to 607 days). The type of CSF complication was related to the type of flap reconstruction used. Out of 8 patients with preoperative history of a VP-Shunt undergoing local axial flap reconstruction, 5 patients suffered from shunt dysfunctions or shunt infections after the local axial flap reconstruction (62.5% of the shunt patients and 33% of all patients treated with axial local flaps). In the patient group undergoing free flap transfer, 8 patients developed new CSF disturbances which were not present before the operative treatment (42.1%); patients were treated for hydrocephalus via VP Shunt (*n*: 3), Omaya reservoir (*n*: 1), external ventricular drain (EVD, *n*: 3), or lumbar drain (*n*: 1). Ten patients suffered from ventriculitis (27%). The 3 patients obtaining the EVD were planned for VP Shunt, but died before their operative treatment of the hydrocephalus. Correlation between the occurrence of CSF infection and CSF disturbance with the need for VP Shunt placement or revision did not reach statistical significance (*r*: 0.278, *p* = 0.096). Sex and age did not influence the onset of CSF disturbances (*p* = 0.527 and *p* = 237). The presence of a previously implanted VP shunt significantly increased the risk for a CSF infection (*r*: 0.536, *p* = 0.001) but not CSF disturbances (*r*: 0.102, *p* = 0.540).

Four patients died shortly after the scalp reconstruction due to postoperative intracranial hemorrhage, acute hydrocephalus, pulmonary artery embolism, or persistent ventriculitis (Table 3).

Mean length of hospital stay was 37 days (range 3 to 120 days).

## Discussion

### General aspects

Complex cranial composite defects present challenging complications for routine neurosurgical procedures. In cases of scalp and skull deficiency, different approaches can be used to perform a scalp reconstruction. In our department, patients are treated by an interdisciplinary team to provide a favorable neurological outcome from the neurosurgical point of view and wound closure and reconstruction from the aspect of reconstructive surgery. As previously described in the results section, postoperative complications did not only include wound-healing difficulties but also neurological complications such as hydrocephalus with the need of VP shunting. Depending on the complexity of the scalp and scull defect, different approaches with varying extent of surgery duration and size are used to achieve skin coverage, and to assure its integrity during adjuvant treatment [9]. We presented a large case series with complex composite defects and focus not only on the reconstructive aspect of the treatment, as most publications do [3, 8, 20, 23], but point out neurosurgical complications influencing the overall outcome. We therefore aim to emphasize the importance of the interdisciplinary setting and draw specific attention to the necessity of neurological monitoring after reconstructive surgery (Table 4).

**Table 4:** Characteristics of the 8 patients developing a new hydrocephalus after reconstructive treatment with age, sex, initial treatment (GBM: glioblastoma, SAH: subarachnoid hemorrhage), presence of a prior ventriculoperitoneal shunt (VP), surgery duration, and LOH (length of hospital stay)

Age	Sex	Initial diagnosis	Presence of VP Shunt?	Surgery duration (min)	Contact flap/dura	Prior surgeries (*n*)	Reconstructive treatment	Surgery	LOH (days)	CSF infection	Death	Time to hydrocephalus (days)
70	0	GBM	0	159	1	2	Vastus lateralis	simultaneous	13	No	1	1
46	1	Astrocytoma	0	359	1	2	Radial	staged	62	No	0	607
55	1	Meningioma	0	415	0	4	Radial	simultaneous	23	Yes	0	6
26	0	Meningioma	0	218	0	4	Lat. dorsi	simultaneous	82	Yes	0	19
74	0	Meningioma	0	555	1	3	Lat. dorsi	simultaneous	55	No	1	7
28	0	SAH	0	300	0	2	Parascapular	simultaneous	53	No	0	42
57	0	GBM	0	320	1	4	Parascapular	simultaneous	53	No	0	6
52	0	Stroke	0	554	0	6	Parascapular	simultaneous	8	No	1	6

### Study limitations

#### Complications

Due to the complexity of simultaneous surgical treatment, the complication rate is high compared to standard flap reconstruction in non-neurosurgical patients. Complication rates usually rise to 15% [19], but do not focus on neurosurgical patients with skull defects and include patients with local skin tumors without intracranial involvement. Scalp deficiencies occurring postoperatively in neurosurgical patients reported higher complication rates than patients treated for local scalp deficiencies [18]. Scalp deficiencies occurring postoperatively in neurosurgical patients present a unique entity. Difficulties, such as infections due to plates and screws after cranioplasty [14], CSF circulation, wound infections due to dead space [16], and radiation therapy before or after the reconstructive surgery [1, 24] have been described.

The published literature mostly includes small case series with few patients treated via a distinct reconstructive approach [6, 7]. The outcome reported in current literature aims to describe satisfying microvascular free flap reconstructive success rates, but neurological complications are rarely described. Large case series focusing on the interdisciplinary approach and neurosurgical complications are underrepresented in the current literature.

The risk of treatment failure, focusing on neurosurgical complications such as CSF disturbances with influence on microvascular free flap acceptance has been reported previously in one case report in 2017 [10]. Here, the negative pressure gradients cause substantial flap sinking or melting. Flap failure was indeed observed in our cohort in 5 cases, but not directly related to CSF disturbances. To our knowledge, complications related to CSF circulation disorders have otherwise not been reported yet.

In total, 8 patients developed a hydrocephalus after scalp and skull reconstruction with free flap transfer, while patients with local axial flaps had complications with preexisting shunts such as clotting or sudden changes in pressure gradients but did not develop a new hydrocephalus. Patients undergoing reconstructive surgery for complex composite defects are indeed at an increased risk of deep postoperative infection and CSF leaks [21], but the new onset of CSF disturbances with the need of surgical treatment and VP-shunting have not been described so far. Long-term outcomes and neurosurgical pathologies are often underreported in studies on reconstructive techniques, and recurrent CSF leaks may be correlated to undetected hydrocephalus, but we lack detailed information in current literature.

Unfortunately, long-term results including the neurological outcome and ventricle width are often understated in publications, and studies report only on flap loss or wound revisions [27]. After an extensive search, we did not find any literature on CSF disturbances and reconstructive techniques in skull and scalp deficiencies, or information on the placement and need of external ventricular drains. Also, information on the occurrence of ventriculitis with antibiotic administration is not mentioned in manuscripts focusing on the success of scalp and skull reconstruction.

Unfortunately, we did not identify the underlying cause of a new hydrocephalus onset in patients undergoing skull reconstruction. Several authors discussed possible etiologies leading to CSF resorption problems. A suggested cause could be an impairment of the glymphatic system, ventricular squeezing, or reduced arterial pulsations due to arachnoiditis or arteriopathy [2, 5, 17]. Unfortunately, we did not perform specific MRI imaging or metabolic analysis to test for the described hypothesis.

### Limitations

Our study was conducted retrospectively; therefore, the causality of complications and their underlying causes can be described, but not proven. We observed many CSF disturbances after reconstructive free flap transfers, pre-existing symptoms of hydrocephalus were not described in the medical data, and preoperative imaging did reveal any signs of CSF disturbances. We presume patients did not suffer from hydrocephalic symptoms, but can retrospectively not exclude it. Chronic wound healing disorders may be caused by pre-existing occult CSF disturbances unapparent on preoperative imaging. The causality between CSF disturbance and reconstructive free flap transfer and which is preceding the other may be different than suspected. As cause and effect cannot be defined retrospectively, we hereby only aim to point out the existence of CSF disturbances after reconstructive surgery, with focus on neurological observation after surgery.

Patients underwent different types of reconstructive surgery, depending on size and complexity of the composite defect. All patients were treated in an interdisciplinary way, with preoperative consultation of both disciplines. In our department, patients remain under the observation of neurosurgeons on a neurosurgical ward focusing on neurosurgical symptoms. Therefore, the detection rate and suspicion of CSF disturbances may be higher than in departments with patients treated for reconstructive surgery without neurosurgical participation. As patients are treated interdisciplinary, the surgical reconstructive treatment and neurosurgical complications can be observed during the same hospital stay. In hospitals with strictly separated departments, the detection of neurological symptoms may be delayed or even missed.

## Conclusion

Neurosurgical complications such as CSF leaks or circulation disorders are common in patients undergoing cranial reconstructive therapy. Especially in patients undergoing free flap reconstruction, CSF disturbances occurred in many cases and must be considered and controlled postoperatively. In order to prevent postoperative complications to assure the optimal neurosurgical and reconstructive outcome, an interdisciplinary treatment needs to be provided for patients with large composite scalp and skull defects.

## Data Availability

The datasets used and/or analyzed during the current study are available from the corresponding author on reasonable request.
